# Supplementary material for: MetaRibo-Seq measures translation in microbiomes
Source: Nat Commun. 2020 Jun 29;11:3268. doi: 10.1038/s41467-020-17081-z (PMC7324362; doi:10.1038/s41467-020-17081-z)
Supplement: Supplementary file 10 — Supplementary Data 7 [file 41467_2020_17081_MOESM10_ESM.zip › File2/Confidence_High_Taxonomy/36774_out.krona.html]

Javascript must be enabled to view this page.

members
magnitude
magnitudeUnassigned
count
unassigned
taxon
rank

36774\_out

3

2
3
superkingdom

phylum
3
1239

class
3
186801

186802
3
order

2
186803
family

572511
1
genus

species

SRS148159\_contig\_number\_51496
1
2292967


SRS143070\_contig\_number\_8166
species
1
1262985

31979
1
family

1485
1
genus

1262792
1
species

SRS017521\_contig\_number\_contig-100\_49247.95505
